# P109: Evaluation of the tolerability and acceptability of alcohol-based hand rub for hand hygiene at Fann Hospital

**DOI:** 10.1186/2047-2994-2-S1-P109

**Published:** 2013-06-20

**Authors:** F Djiby, Bara Ndiaye, Mery Dia

**Affiliations:** 1 Fann Hospital, Dakar, Senegal

## Introduction

The use of alcohol-based hand rubis an indirect marker of the effective implementation of hand hygiene. Caregivers are in direct contact with patients on a daily basis and use thealcohol-based hand rub during health care. Discomforts of use of this product seem to be reported by some practitioners.

## Objectives

That is why we are interested in evaluating the tolerability and acceptability of the product at Fann Hospital in the exposed workers.

## Methods

Among caregivers, 40 using the alcohol-based hand rubfor hand hygiene routine for at least 30 days were subjected to a questionnaire. Approximately 10 minutes were necessary to complete the questionnaire. A subjective assessment by the participant on risk factors for skin lesions, product acceptability and tolerance was thus achieved through the questionnaire.

## Results

Nodermalaggressionwas reported byinterviewed caregivers.Only 5of the 40caregiversexposedreportedunpleasant odorof alcohol-based hand rub. All caregiversinterviewed reportedeasy useof the alcohol-based hand rub.

## Conclusion

Alcohol-based hand rubsare acceptable andwell toleratedby practitionersduringnormal use.However, therevision of theWHOformulafor making theproductshould be consideredin the context ofinternational conferences toimprove the smellof the product, a factor limiting its use.

## Disclosure of interest

None declared

